# Nanoparticle-based drug delivery systems to enhance cancer immunotherapy in solid tumors

**DOI:** 10.3389/fimmu.2023.1230893

**Published:** 2023-08-03

**Authors:** Jiaxin Zhang, Siyuan Wang, Daidi Zhang, Xin He, Xue Wang, Huiqiong Han, Yanru Qin

**Affiliations:** ^1^ Department of Oncology, The First Affiliated Hospital of Zhengzhou University, Zhengzhou, China; ^2^ Department of Pathology, The First Affiliated Hospital of Zhengzhou University, Zhengzhou, China; ^3^ Academy of Medical Science, School of Basic Medical Science, Zhengzhou University, Zhengzhou, China

**Keywords:** nanodrug delivery systems, nanoparticles, immunogenic cell death, immunotherapy, tumor microenvironment, solid tumors, EPR effect

## Abstract

Immunotherapy has developed rapidly in solid tumors, especially in the areas of blocking inhibitory immune checkpoints and adoptive T-cell transfer for immune regulation. Many patients benefit from immunotherapy. However, the response rate of immunotherapy in the overall population are relatively low, which depends on the characteristics of the tumor and individualized patient differences. Moreover, the occurrence of drug resistance and adverse reactions largely limit the development of immunotherapy. Recently, the emergence of nanodrug delivery systems (NDDS) seems to improve the efficacy of immunotherapy by encapsulating drug carriers in nanoparticles to precisely reach the tumor site with high stability and biocompatibility, prolonging the drug cycle of action and greatly reducing the occurrence of toxic side effects. In this paper, we mainly review the advantages of NDDS and the mechanisms that enhance conventional immunotherapy in solid tumors, and summarize the recent advances in NDDS-based therapeutic strategies, which will provide valuable ideas for the development of novel tumor immunotherapy regimen.

## 1 Introduction

**Figure 1:**
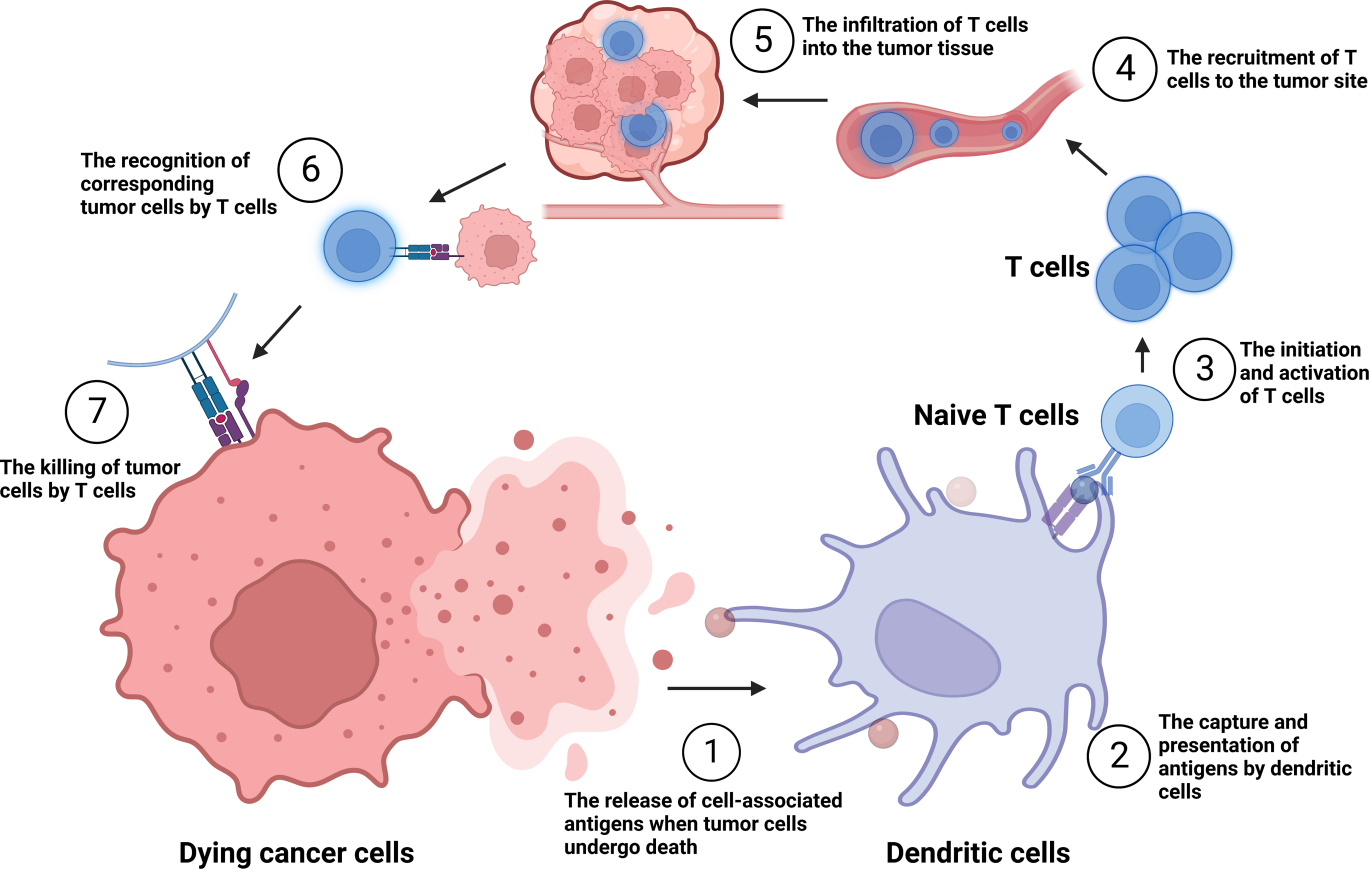
The tumor immune cycle put forward by Chen and Mellman. ①-⑦: the release of cell-associated antigens when tumor cells undergo death; the capture and presentation of antigens by dendritic cells (DCs); the initiation and activation of T cells; the recruitment of T cells to the tumor site; the infiltration of T cells into the tumor tissue; the recognition of corresponding tumor cells by T cells; and the killing of tumor cells by T cells.

Since 2013, Chen and Mellman have proposed the concept and critical mechanisms of the tumor immune cycle, suggesting the importance and potential promise of immunotherapy (**Figure 1**). Specific mechanisms include: a) the release of cell-associated antigens when tumor cells undergo death; b) the capture and presentation of antigens by dendritic cells (DCs); c) the initiation and activation of T cells; d) the recruitment of T cells to the tumor site; e) the infiltration of T cells into the tumor tissue; f) the recognition of corresponding tumor cells by T cells; and g) the killing of tumor cells by T cells via the secretion of granzyme, perforin, and other effectors (1). Immunotherapy has received more attention in recent years due to its robust efficacy and tolerable toxicity. According to the tumor immune cycle, current tumor immunotherapy mainly comprises immune targets antibody, tumor vaccine, cellular immunotherapy, oncolytic virus, and cytokine therapy (2). Chemotherapy drugs are also commonly used to enhance tumor cells immunogenicity, affect the function of immune cells such as DCs, Myeloid-derived suppressor cells (MDSCs), and Regulatory T cells (Tregs) (3). Immune checkpoint inhibitors (ICIs) such as PD-1/PD-L1 and CTLA-4 inhibitors, together with chemotherapy, have received satisfactory results in various clinical trials for most solid tumors (4). However, efficacy is limited by immune-related adverse events, therapeutic resistance, high cost, and limited therapeutic patients (5, 6).

Recently, we found that immunogenic cell death (ICD) induction might play a critical factor in improving immunotherapy. ICD releases tumor-associated antigens and endogenous danger signals, further triggering antigen-presenting cells and ultimately activating effective antitumor immune response (7). Several therapies such as chemotherapy, radiotherapy (RT), photothermal therapy (PTT), photodynamic therapy (PDT), and sonodynamic therapy (SDT) and drugs (certain chemotherapeutic drugs, traditional Chinese medicines) have worked to initiate and/or enhance ICD, leading to the activation of tumor-specific immune responses (8, 9). Additionally, the tumor microenvironment (TME) plays a significant role in promoting the tumor immunosuppressive microenvironment (TIME) (10). Based on these points, we need to explore novel methods to overcome current limitations and increase the immunotherapy effect.

One of the novel ways of improving the efficacy of tumor immunotherapy is nanodrug delivery systems (NDDS), which have the features of small size and ease editing. Various smart responsive nanoparticles (NPs) have been designed to deliver drugs accurately to the tumor site, which greatly weakens the “off-target” effect (11–13). Many experiments have shown that NDDS therapy combined with PD-1/PD-L1 antibodies can not only enhance immune efficacy but also control distant metastasis and recurrence of tumors (14). In this article, we mainly discuss the mechanisms of NDDS to enhance immunotherapy and summarize recent NDDS based treatment strategies. We describe the advantages of enhanced anti-tumor effects of NDDS by elucidating the relevant mechanisms. And we summarize the mechanisms of how NDDS-based treatment strategies enhance immunotherapy, including the induction of ICD process and the reprogramming of immunosuppressive microenvironment.

## 2 The status and limitation of immunotherapy in solid cancers

Tumor immunotherapy comprises various strategies, including ICIs, cellular immunotherapy (15), therapeutic tumor vaccines (16), oncolytic viruses (17), and cytokine immunotherapy (18). ICIs are the most widely used strategy for the treatment of solid tumors. Following the FDA approval of Ipilimumab (a CTLA-4 inhibitor) for advanced melanoma in 2011, ICIs quickly gained approval for treating various cancer types (19), as evidenced by several FDA-approved immune checkpoints such as PD-1 and its receptor PD-L1, CTLA-4, and LAG3. CTLA-4 inhibitors (e.g. Ipilimumab and Tremelimumab), PD-1 inhibitors (e.g. Pembrolizumab, Nivolumab, and Cemiplimab) and PD-L1 inhibitors (e.g. Atezolizumab, Durvalumab, and Avelumab) are widely used alone or in combination to treat different types of cancer, including melanoma, non-small cell lung cancer, bladder cancer, renal cell carcinoma, head and neck squamous cell carcinoma, among others (20). Until last year, the second-generation checkpoint inhibitor Opdualag (targeting LAG-3) was just launched, and it was classified as a first-line treatment for unresectable melanoma along with the PD-1 inhibitor nivolumab. This result is based on a phase 2/3 study in which the median progression-free survival with the combination (10.1 months) was stronger than with Opdivo monotherapy (4.6 months), showing promising clinical response (21). Immunotherapy has emerged as a revolutionary approach to treat cancer, but its efficacy is often limited (22). While mild cases of CRS manifest as fever, fatigue, headache, rash, joint pain, and myalgia, severe cases can lead to an uncontrolled systemic inflammatory response characterized by low blood pressure and high fever (23). Although CAR-T therapy has been successful in treating B-cell lymphomas and leukemias, it has shown limited effectiveness against solid tumors, possibly due to low penetration in the tumor matrix, pressure gradients, and the immunosuppressive microenvironment (24). Other immunotherapies such as therapeutic tumor vaccines, oncolytic viruses, and cytokines face major safety, efficacy, and delivery barriers. One of the major limitations is the low number of patients who respond to ICIs despite their breakthrough success, adding to the issue that patients may gradually develop resistance to immunotherapy (20). Furthermore, a significant proportion of patients experience immune-related toxicity, such as cytokine release syndrome and immune effector cell-associated neurotoxic syndrome (25). Improving drug transport ways to enhance drug concentration and targeting ability is currently recognized as an effective method.

## 3 The advantage of nanodrug delivery system in solid tumors

NDDS primarily rely on the encapsulation of drugs or biological molecules within particles with a diameter less than 100 nm. Alternatively, NDDS also encompasses materials within the range of 100 nm to 1,000 nm, but possessing nanoparticle behavior. These systems employ distinctive particle properties such as acoustic, electric, optical, magnetic, and thermal attributes to facilitate the targeted transportation of nanoparticles bearing drugs or other biological molecules to specific cells or tissues. FDA-approved NDDS materials primarily include, but are not limited to liposomes, polymers, and inorganic materials (26). More materials of NPs are being developed.

**Figure 2:**
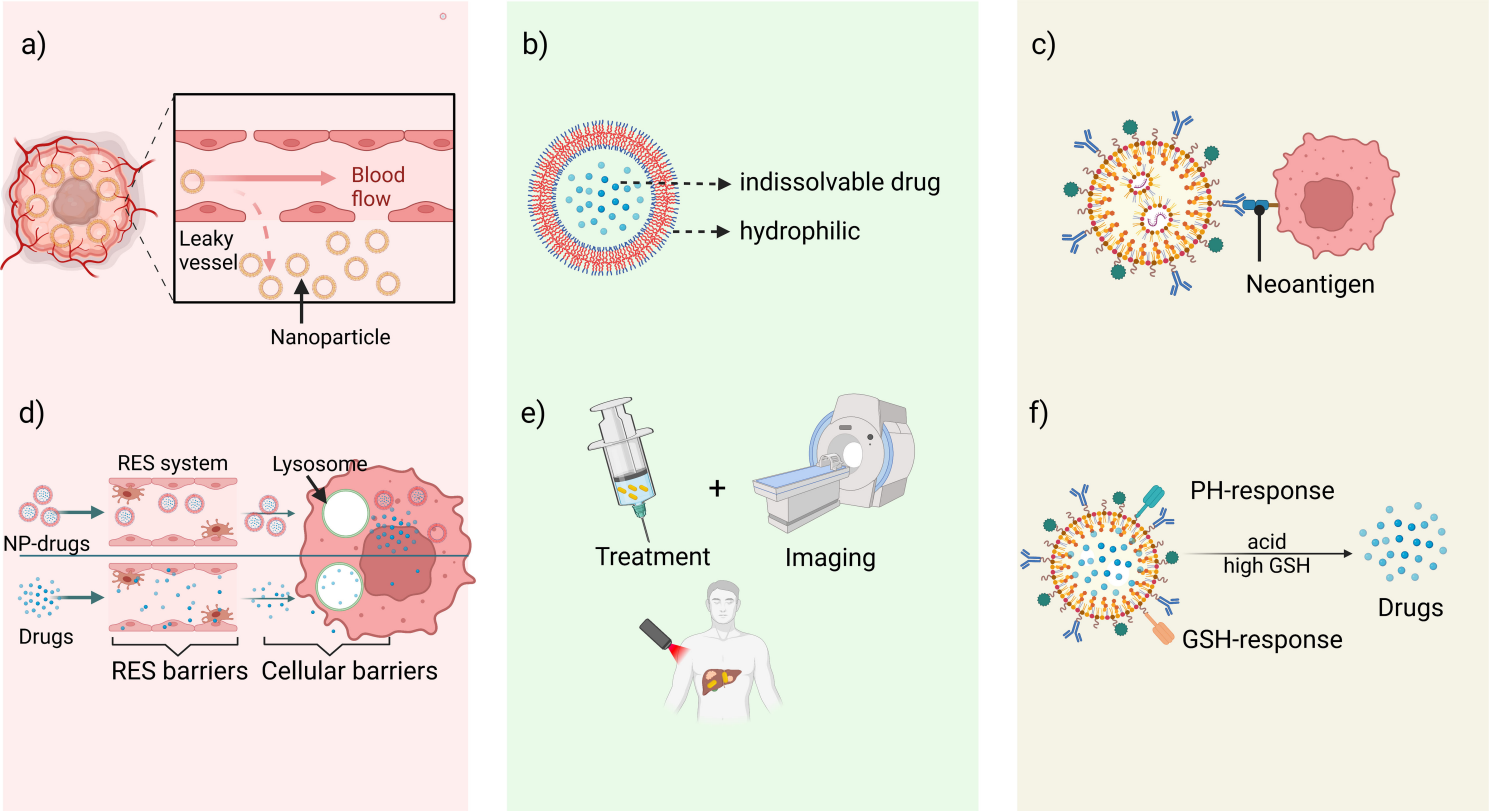
The advantages of NDDS-based immunotherapy. **(A)** Enhanced permeability and retention (EPR) effect. **(B)** Improving solubility of hydrophobic drugs. **(C)** Achieving active targeting delivery by specific antigens. **(D)** Reducing physiological barriers to drug hindrance. **(E)** Integration of diagnosis and treatment. **(F)** Stimulus-responsive intelligent nanodrug delivery systems.

Compared with traditional immunotherapy in solid cancers, NDDS-based immunization strategies provide more advantages (**Figure 2**). They are listed as follows: a) Enhanced permeability and retention (EPR) effect. EPR effect is a passive targeting process that can be induced by the interaction of endothelial lining gaps extravasation and immune cells in the TME (27–29). However, EPR effects are more pronounced in preclinical studies (small animal xenograft tumor models) and very limited in human tumors, which is a very controversial topic for current research (30). b) Improving solubility of hydrophobic drugs. Most clinical drugs have poor water solubility and biocompatibility. However, NDDS overcomes this obstacle in by creating lipophilic cavities internally to encapsulate drugs, while the external structure of the carrier is hydrophilic and better suited to biological tissues (31, 32). c) Achieving active targeting delivery. NDDS can bind to specific receptors on the cell surface using specific ligands or antibody components, to achieve accumulation in the targeted tissue. The information from the overexpression of some genes, cytokines, and proteins of tumor cells forms the basis for the design of targeted drugs containing intelligent recognition sites. Commonly used targeting molecules include antibody fragments and molecules, folic acid corresponding to ligands (33), proteins (e.g. transferrin) (34), peptide molecules (e.g. RGD and TAT) (35, 36), nucleic acid aptamers (e.g. aptamer) (37) and polysaccharides (e.g. mannose) (38). d) Reducing physiological barriers to drug hindrance. Non-NPs-based drug molecules must undergo phagocytosis by the reticuloendothelial system (RES), cell membrane barrier-permeation and intracellular transport barriers-lysosomal degradation before reaching the target. While most NDDS are designed smaller and multifunctional which can evade these obstacles (39). e) Integration of diagnosis and treatment. Nanomaterials have unique physical properties such as light, heat and magnetism, which offer nanocarriers the potential to be used as diagnostic probes with imaging. Monitoring drug concentration at the target site greatly improves therapeutic efficiency (40). f) Stimulus-responsive intelligent nanodrug delivery systems (41). most tumor microenvironment is characterized by hypoxia, acidity, high expression of enzymes, glutathione and reactive oxygen species, based on which single-factor, or multi-responsive nanomaterial delivery systems can be designed to achieve targeted drug delivery (42). Since FDA approved the first liposome nanodrug - Doxil in 1995, many drugs for treating cancers have been launched one after another (**Table 1**).

**Table 1:** The summary of FDA approved nanodrugs in cancers.

Drugs	Drug-loading	Cancers	Approval year	Approved by FDA
**Doxil**	Polyglycolated doxorubicin liposomes	Ovarian and breast cancer	1995	Yes
**Onivyde**	Liposomal irinotecan	Pancreatic cancer	2015	Yes
**Myocet**	Doxorubicin liposomes	Breast cancer	2000	Yes
**MEPACT**	Cell wall acyl tripeptide phosphatidyl ethanolamine liposome	Osteosarcoma	2009	Yes
**SMANCS**	Poly (styrene-co-maleic acid/anhydride)–neocarzinostatin conjugate	Liver and renal carcinoma	1993	Yes
**Genexol-PM**	Paclitaxel micellar formulation	Breast and small cell lung cancer	2007	Yes
**Paclitaxel Liposome for Injection**	Paclitaxel liposome	Breast, lung and ovarian cancer	2003	Yes
**Abraxane**	Albumin-bound paclitaxel nanospheres	Multiple cancers and metastatic pancreatic cancer	2005	Yes
**Eligard**	Lepraline acetate polymer	Prostatic cancer	2002	Yes
**Ryanodex^®^**	Dantrolene sodium	Malignant hypothermia	2014	Yes
**Nano-therm**	Iron oxide	Glioblastoma	2010	Yes

## 4 The mechanisms of nano-drug delivery system to enhance immunotherapy

NDDS were initially designed to alter the toxicity and pharmacokinetics profiles of chemotherapy agents, allowing for higher drug concentrations to accumulate inside tumors. Recently, with the identification of new immune activation pathways, there has been a surge of interest in using nanomaterials for immunotherapy against solid tumors. The main mechanisms of action involve the induction of ICD and modulation of the immunosuppressive microenvironment.

### 4.1 NDDS enhances immunotherapy by inducing immunogenic tumor cell death

**Figure 3:**
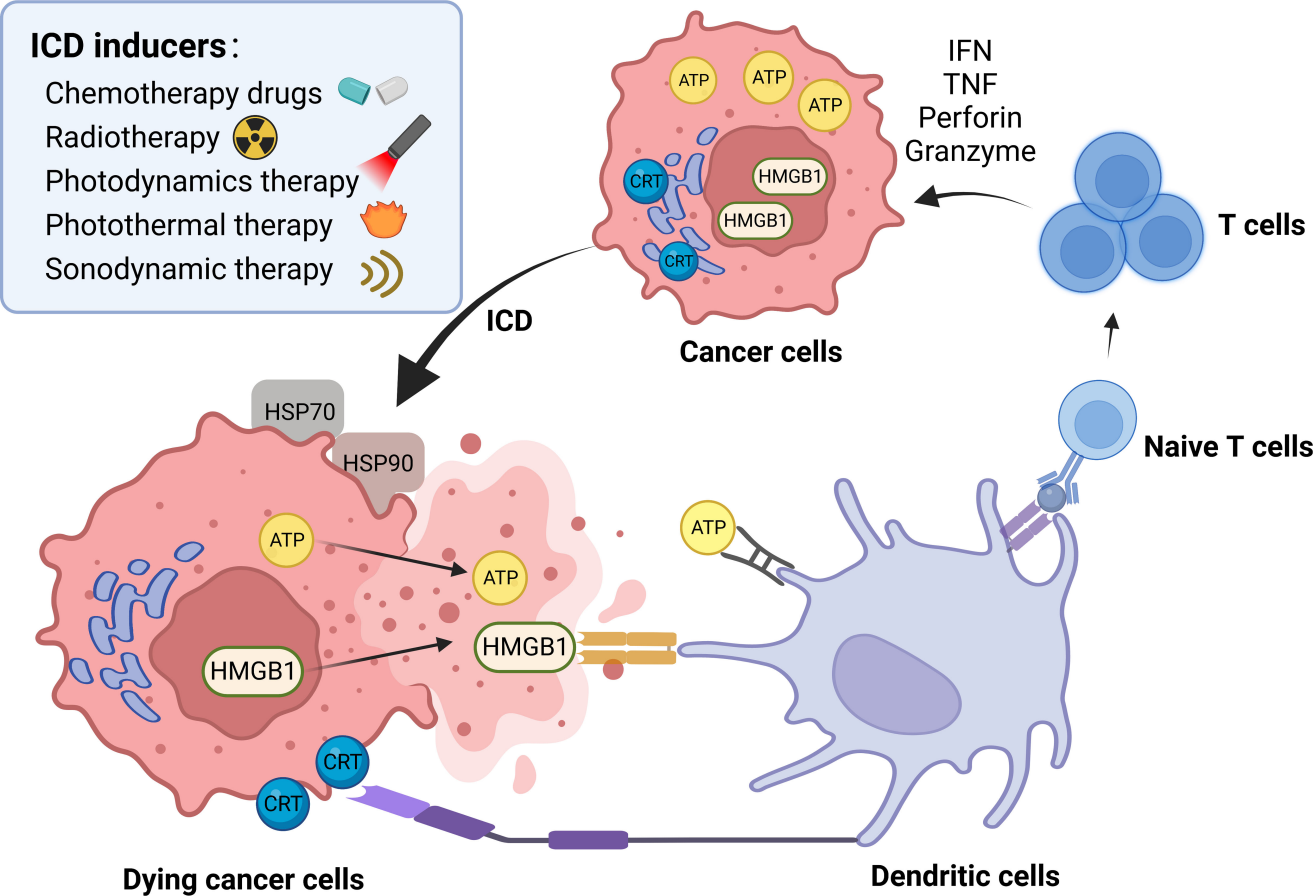
ICD process and common inducers. DAMPs are the main pattern of ICD process, including calreticulin (CRT) that exposes on the cell surface, secreted ATP, heat shock proteins (HSP70 and HSP90), and high mobility group protein 1 (HMGB1). Some chemotherapy drugs, radiotherapy, photodynamic therapy, photothermal therapy and sonodynamic therapy are common ICD inducers. → represents the pointing and promoting effect.

**Figure 4:**
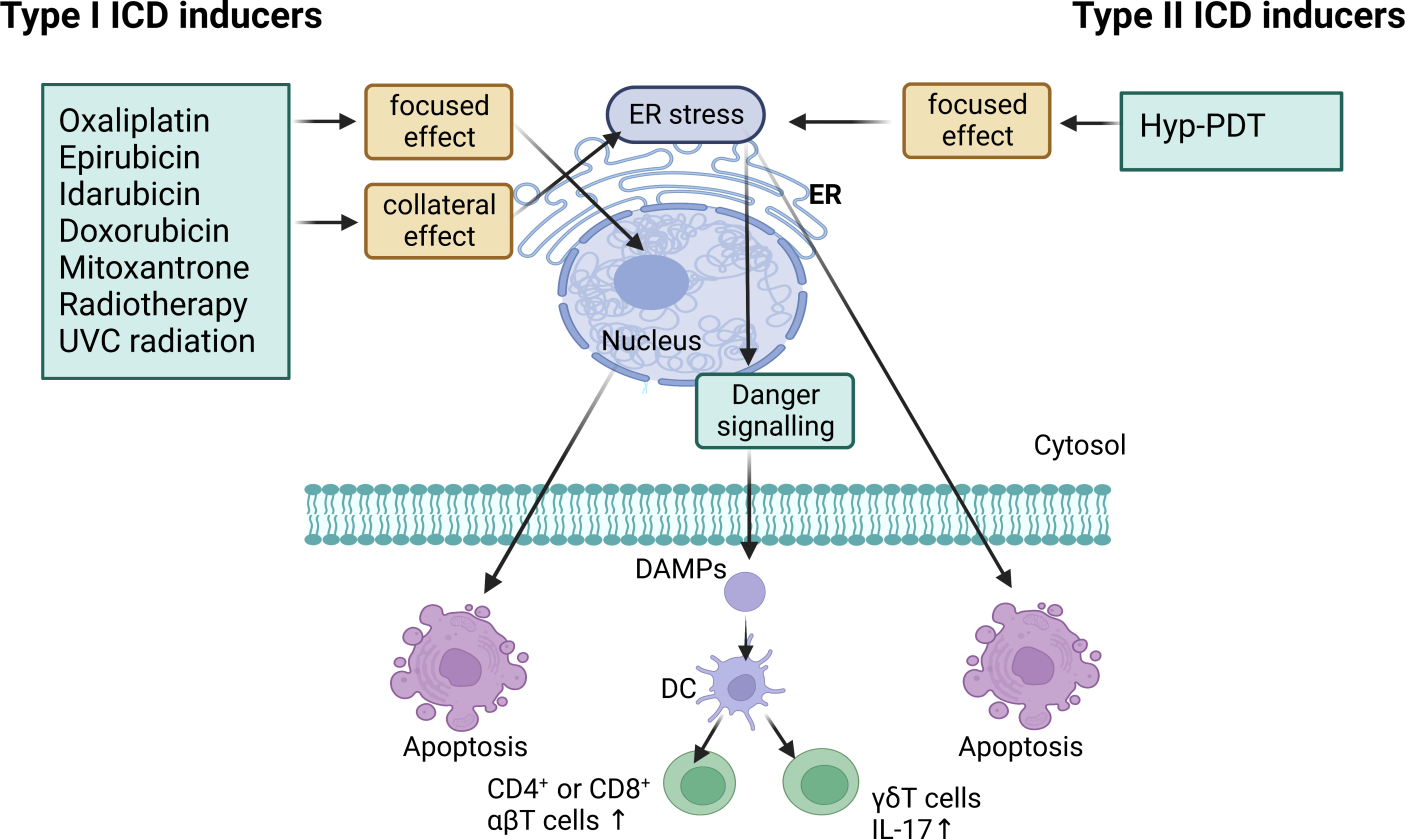
Type I ICD inducers are anticancer drugs that operate on non-ER proteins to induce cell death while also promoting collateral ER stress for danger signaling. Type II ICD inducers are anticancer agents that target the ER for both cell death induction and danger signaling. They both have the ability to induce the emission of damage-associated molecular patterns (DAMPs) which cause the activation of IL-17-producing γδT cells and increased proliferation of CD4^+^ or CD8^+^ αβT cells. Hyp-PDT, hypericin-based photodynamic therapy.

Cell death stimuli in tumors induce tumor cells to become immunogenic, resulting in the release of molecules that initiate an anti-tumor immune response called ICD process (43). Throughout this process, the tumor cells produce a group of signaling molecules known as damage-associated molecular patterns (DAMPs) (44), including calreticulin (CRT) that exposes on the cell surface, secreted ATP, heat shock proteins (HSP70 and HSP90), and high mobility group protein 1 (HMGB1) (**Figure 3**). Anthracyclines and oxaliplatin stimulate calreticulin to transfer from the intracellular site to the outer surface of the cell membrane where it is recognized by DCs and presented as tumor antigens, thus initiating the ICD process (45). Other chaperone molecules such as HSP70 and HSP90 can also valgus to the surface of the cell membrane as “eat me” signals (46). The release of ATP serves as a “find me” signal, that attracts mature or immature DCs close to the tumor site, delivering the energy needed for DCs to present antigens (47, 48). HMGB1 acts as the most critical initiator of ICD by binding to Toll-like receptor 4 on DC membranes, thereby activating cytotoxic T cell lymphocytes (CTLs). ICD inducers are commonly categorized into two classes known as type I and type II. Type I ICD inducers initiate the apoptosis of cancer cells proficiently, without affecting the endoplasmic reticulum (ER). These ICD inducers generate a moderate level of ER stress and discharge DAMPs that result in the formulation of immunogenic molecules. Conversely, type II ICD inducers produce more significant ER stress along with elevated levels of reactive oxygen species (ROS), and discharge more DAMPs, ultimately escalating the tumor’s immune response to a greater extent (49, 50). **Figure 4** depicts the classification mechanisms of the currently established ICD inducers (51, 52). However, not all clinical drugs or therapeutic strategies can activate ICD, nor can all ICD inducers be efficiently utilized. The use of NDDS provides a new and efficient approach for boosting the existing therapeutic efficacy and reducing the side effects of cancer agents (53). **Table 2** outlines the present clinical ICD inducers and immunotherapeutic strategies. In this section, we will discuss how NDDS-based therapeutic strategies can enhance immunotherapy by inducing ICD.

**Table 2:** The summary of ICD inducers and immunotherapeutic strategies.

Strategy	Agent	Nanoparticle	Immune effect	Cancer type	Cell lines	Mouse models	Publication year	Ref
**Nano-based chemo-immunotherapy**	Fluorouracil + oxaliplatin + anti-PD-L1	Nano-Folox/Nano-FdUMP	Increased CD8+ T cells, CD4+ T cells, dendritic cells, IFN-γ, TNF-α and IL-12 and decreased MDSCs, Tregs, M2 macrophages, IL-4, IL-6 and IL-10	CRC and HCC	CT26 Hepa1–6	*in situ* CRC and HCC mice	2021	(54)
	Ginsenoside Rg3 + quercetin (QTN) + Anti-PD-L1	D-PEG-FA.Rg3.QTN	Increased CD8+ T cells, CD4+ T cells and activated DCs, IFN-γ, IL-12, CXCL9 and CXCL10; Decreased Tregs, MDSCs, M2, IFN-γ, IL-12, CXCL9 and CXCL10	CRC	Mouse CT26 and human HCT116 CRC cell lines	orthotopic CRC mouse model	2022	(55)
	Oxaliplatin and NLG919	DDP NPs	Promoting the maturation of DCs, the infiltration of cytotoxic CD3+CD8+ T lymphocytes, the secretion of TNF-α, IFN-γ, and IL-2 and decreased Tregs	CRC BC	CT26	colorectal tumors, breast tumors,	2022	(56)
	Irinotecan+ anti-PD-1	Mesoporouss silica nanoparticle	Increased CD8+/Treg ratio and decreased Tregs	PDAC	KPC cells	PDAC mouse model	2021	(57)
	DOX+ anti-PDL-1	DOX@DCS	PD-1/PD-L1 blockade	CRC	CT26 and 4T1	CT26 cell mouse model	2021	(58)
	JQ1 + doxorubicin	Orchestrated yolk-shell nanoparticle system	Caspase 3 activation, and cytotoxic T lymphocyte infiltration	melanoma carcinoma	B16-F10	mice bearing B16-F10 tumor xenograft	2023	(59)
	DOX + ATP Fe2 + PD-L1	PADO-Fe	Induce phenotype transition of TAM from M2 to M1; generate O2 and hydroxyl radical	BC	4T1	4T1 tumor-bearing mice	2022	(60)
	Epirubicin+ STF-62247	STF@AHPPE nanoparticles	Increased matured DCs, cytotoxic T cells, TNF-α, IFN-γ, IL-6 and enhanced autophagy	CRC	CT26	CT26 tumor-bearing mice	2023	(61)
	Bortezomib	PHDZ/BTZ nanoparticles	Increased CD8+ cytotoxic T cells, IL-6, TNF-α, and IFN-γ and reduced TGF-β	BC	4T1	4T1 tumor-bearing mice	2022	(62)
	Bortezomib	PAG/BTZ nanoparticles	Activated DC maturation, increased IFN-γ and IL-6 and decreased TGF-β	BC	4T1	4T1 tumor-bearing mice	2023	(63)
**Nano-based RT/PDT/PTT/SDT**	Cisplatin+RT	CDDP-NPs	Increased CXCL10 and CD8+ T cell	lung carcinoma	LLC	LLC tumor model	2021	(64)
	AuNPs+RT	AuNPs	Increased macrophage infiltration	TNBC	MDA-MB- 231	xenograft mouse model	2021	(65)
	ZGd-NRs+anti-PD-1+RT	ZGd-NRs	Increased DCs maturation, CD8+ T cells and decreased IL-10, VEGF-A	CRC	CT26	4T1 tumor-bearing mice	2021	(66)
	Oxaliplatin+ NTKPEG	OPCPN@NTKPEG	Stimulated CRT exposure, promoted DCs maturation, increased CTLs, and reduced Tregs	BC	4T1	4T1 tumor-bearing mice	2021	(67)
	Glutathione+ RT	MGTe	Promoted DCs maturation, increased IL-12, TNF-α, IL-6, IL-12, M1 macrophages, CD3+CD8+ T cells and IFN-γ	BC	4T1	4T1 tumor-bearing mice	2022	(68)
	(D)PPA-1 + R848	NIA-D1@R848 nanoparticles	Promoted DCs maturation; increased CTL infiltration, CD8+T cells, TAMs, IL-2 and IFN-γ	colon cancer	MC38	MC38 tumor-bearing mouse	2022	(69)
	Multifunctional black phosphorus+PDT/PTT	HA-BP nanoparticles	Promoted DCs maturation; increased IL-2, CD4+ and CD8+ T cells and decreased IL-10	BC	4T1	4T1 tumor-bearing mice	2021	(70)
	Axitinib+ Ce6 + 1MT	NIR-ratiometric nanoplatform (PTP)	Increased CD8+ T cells, TNF-α and IFN-γ	BC	4T1	4T1 tumor-bearing mice	2021	(71)
	AuNC@MnO2 +PDT	AuNC@MnO2, AM)	Promoted DCs maturation, increased CD8+CD69+ T cells, CD4+CD69+ T cells and NK1.1+CD69+ cells, and decreased Tregs	TNBC	4T1	4T1 tumor-bearing mice	2018	(72)
	Polydopamine-Doxorubicin+PDT	PDA-DOX NPs	-	Renal Cancer	HK-2 OS-RC-2/ADR	–	2021	(73)
	CAT+DTA-1+PDT/PTT	PDA-ICG@CAT-DTA-1	Increased CD4+ effector T cells and decreased Tregs	BC	4T1	4T1 tumor-bearing mice	2021	(74)
	Oxaliplatin+indocyanine green+ PTT	metal-organic framework MIL-100 (Fe) nanoparticles (NPs)	Increased CD4+ T cells and CD8+ T cells	CRC	CT26 3T3	CT26 tumor-bearing mice	2022	(75)
	Nano-zirconia+PTT	ZrO2-x@PEG/cRGD	Increased IFN, TNF-α and IL-6	BC	4T1	4T1 tumor-bearing mice	2021	(76)
	HA-AuNR+PTT	HA-AuNR/M-M2pep NP	Increased TILs, IFN-γ and TNF-α	melanoma	B16F10	B16F10 tumor bearing mice	2021	(77)
	Tic+PTT	DPC@ICD-Gd-Tic	-	BC	4T1	4T1 tumor-bearing mice	2021	(78)
	AuDRM+PTT	Dendritic mesoporous silica nanoparticles (NPs)	Promoted DCs maturation, increased effector memory T (TEM) cells	BC	4T1	4T1 tumor-bearing mice	2021	(79)
	R837+PTT	HA-PANi/R837 NPs	Increased CTL, TNF-α and IFN-γ	TNBC	MDA-MB-231 4T1	4T1 tumor-bearing mice	2021	(80)
	IR780+ PFH_SDT	IRO@FA np	Increased CD3+ T and CD8+ T cells, IL-6, TNF-α, IFN-γ and PD-L1	EOC	ID8	ID8 ovarian cancer bearing mice	2022	(81)
	Docetaxel (DTX) +SDT	CS-Rh-PFC	Enhanced secretion of IFN-γ, TNF-α, IL-2 and IL-6 cytokines and tumor-infiltrating CD4+ and CD8+ T cells	melanoma	B16F10	B16F10 tumor bearing mice	2021	(82)
	FAMnPs+ SDT	FA-MnPs	Re-polarizes immunosuppressive M2 macrophages to antitumor M1 macrophages, and activate DCs, T lymphocytes, and NKs	BC	4T1	4T1 tumor-bearing mice	2021	(83)
	Oxaliplatin+ SDT	OXI-NPs	-	EOC	ID8	ID8 ovarian cancer bearing mice	2021	(83)
**Nano-based gene editing**	IDO1 siRNA	NPs	Promoted DCs maturation, increased tumor-infiltrating T lymphocytes and decreased Tregs	CRC and PAAD	CT26 Panc02	CT26 tumor-bearing mice Panc02 tumor-bearing mice	2019	(84)
	IDO1 siRNA and mitoxantrone	Acidity-triggered charge-reversal NPs	Promoted DCs maturation, increased CTLs and decreased Tregs.	BC and CRC	4T1 CT26	4T1 tumor-bearing mice CT26 tumor-bearing mice	2022	(85)
	Nrf2-siRNA	TIR@siRNA	Improved cytotoxicity of SDT and induced ICD.	CRC	CT26	CT26 tumor bearing mice	2021	(86)
	PD-L1 siRNA	CbP/siPD-L1@Dig	Increased CD3ϵ+CD4+ Helper T cells (Ths) and CD3ϵ+CD8a+ Cytotoxic T cells (Tcs)	CRC and OC	CT26, MC38, and ID8	CT26 tumor-bearing mice MC38 tumor-bearing mice ID8 tumor-bearing mice	2021	(87)

CRC, colorectal cancer; BC, breast cancer; TNBC, Triple Negative Breast Cancer; OC, ovarian cancer; PAAD, Pancreatic Acinar Cell Carcinoma; EOC, epithelial ovarian cancer; PDAC, Pancreatic Ductal Adenocarcinoma.

“-” means none.

#### 4.1.1 Nano-based chemo-immunotherapy

Firstly, compared to non-ICD inducers, ICD inducers demonstrate greater potential in antitumor treatment in clinical settings. Chemotherapy drugs such as doxorubicin, epirubicin, idarubicin, mitoxantrone, bleomycin, bortezomib, cyclophosphamide, and oxaliplatin can induce ICD if used as solitary therapeutic interventions (49). However, these drugs may have weak immunogenicity and lead to adverse effects for many patients. NDDS can ameliorate these problems by extending the functioning cycle of chemotherapy drugs with a reverse-phase protein array effect, minimizing toxic side-effects by utilizing precise targeting characteristics, and augmenting tumor immunity with ICD, thus opening new opportunities for combination chemotherapy and immunotherapy (88, 89).

Nanoparticles contribute to both ICD induction and microenvironment reprogramming. For example, Fluorouracil (5-Fu) and oxaliplatin (OxP) are two commonly used chemotherapeutic agents for CRC and HCC and both are ICD inducers (51, 90). FOLFOX (Folinic acid + 5-Fu + OxP) is a standard chemotherapy regimen for advanced CRC and HCC patients. Jianfeng Guo et al. designed two nano-formulations to treat *in situ* CRC and HCC mice with or without PD-L1 antagonists. One Nano-formulation was nano-FdUMP (5-Fu active metabolite), which induced ROS formation and significantly improved the efficacy of the second nanoformulation, nano-Folox, in inducing ICD. However, nano-FdUMP could not induce ICD on its own but achieved an efficient chemo-immunotherapeutic response in combination with nano-Folox. The combination of both nanoformulations transformed the ‘cold’ TME into a ‘hot’ one, supported by increased numbers of CD8+ T cells, CD4+ T cells, dendritic cells, IFN-γ, TNF-α, and IL-12 and reduced numbers of MDSCs, Tregs, tumor-associated macrophages (M2), IL-4, IL-6, and IL-10. With the immunosuppressive TME reprogrammed, the anti-PD-L1 monoclonal antibody showed improved efficacy against microsatellite stable CRC liver metastasis (54). Bing Feng’s team designed a binary cooperative prodrug nanoparticle (BCPN) that, when triggered by an acidic environment, OxP and NLG919 (an IDO-1 inhibitor that regulates immunosuppression) were activated and released, achieving a stronger EPR effect, promoting T lymphocyte infiltration by stimulating ICD recruitment, and reducing tumor burdens (breast and colorectal cancer) (91). 3-(2-nitrophenyl) propionic acid-paclitaxel nanoparticles (NPPA-PTX NPs) work as an ICD inducer in MDA-MB-231 and 4T1 cell lines by upregulating HMGB1 and CRT. The combination of NPPA-PTX nanoparticles and anti-PD-L1 monoclonal antibody enhances the antitumor response by recruiting infiltrating CD8+, CD3+, CD4+ T cells and increasing IFN-γ and TNF-α levels (92). While irinotecan is a weakly alkaline drug that neutralizes the acidic lysosomal environment in pancreatic ductal adenocarcinoma (PDAC) cells. Based on this, Mesoporous silica nanoparticles delivery system amplifies this advantage, causing a series of endoplasmic reticulum response, immunogenic cell death, and PD-L1 expression. This effect was observed in an orthotopic Kras-dependent pancreatic cancer model. When combined with anti-PD-L1 therapy, the use of silicosomes showed better chemo-immunotherapy response compared to free or liposomal drugs such as Onivyde (57). Chemotherapeutic agents may also induce immunogenic cell death through autophagy process to mediate antitumor immunotherapy. A recent study of STF@AHPP nanoparticles, combining epirubicin with an autophagy inducer STF-62247 have shown a stronger immune activation ability than epirubicin alone in mice with CT26 tumors. This provides a new approach to combining chemo-immunotherapy and autophagy agonist (61).

Of course, in addition to the regular chemotherapeutic agents, there are new drugs that can also induce ICD and contribute to chemo-immunotherapy. Ginsenoside Rg3 and quercetin (QTN) are examples of a novel ICD inducer and a ROS generator, which enhance the Rg3-mediated ICD. A folate-targeted PEGylated cyclodextrin-based nanoparticle that delivers Rg3 and QTN was developed by Dandan Sun et al. This co-delivery formulation, when combined with anti-PD-L1 antibody, achieved chemo-immunotherapy in colorectal cancer patients who were insensitive to ICIs (55). Chinese herbal medicine-Icariin was encapsulated by polylactic acid-glycolic acid and injected into gastric cancer patients as PLGA@Icaritin NPs. PLGA@Icaritin may also contribute to the production of dozens of ROS, which can result in a significant loss of mitochondrial membrane potential and overproduction of oxidized mitochondrial DNA (Ox-mitoDNA). This leads to the release of DAMPs and ultimately results in ICD. *In vivo* studies have shown that PLGA@Icaritin nanoparticles enhance anti-tumor immunity by recruiting infiltrating CD4+ cells, CD8+ T cells, and immune factors such as IFN-γ, TNF-α, and IL-1 (93).

#### 4.1.2 Nano-based RT/PDT/PTT/SDT

Ablative treatments for cancer, including radiotherapy (RT) (94), photodynamics therapy (PDT) (95), photothermal therapy (PTT) (96) and sonodynamic therapy (SDT) (97) are effective methods for inducing ICD in various solid tumors. Often acting as type II ICD inducers, ablative therapies generate ROS and stimulate ER stress response (98). Different nanomaterials can be employed to transport medications, vaccines and other compounds to the tumor location for augmented drug accumulation in the radiation treatment zone, resulting in elevated sensitivity of RT/PDT/PTT/SDT. At the same time, NDDS with acoustic, optical, thermal, and magnetic properties can be used to realize imaging tacking *in vivo*, providing convenience for the precision of immunotherapy (76, 99, 100).

RT has been extensively used in clinical practice as a local therapy for more than a century. Although RT mainly targets the DNA of tumor cells, the induction of ICD provides a new possibility for immunotherapy (101). In fact, RT is a dose-dependent therapy. Large doses of radiotherapy primarily trigger ICD, release tumor-specific antigens, activate immune cells, and enhance the density of tumor-infiltrating lymphocytes (TILs). Low doses of RT primarily recruit immune cells, regulate the inflammatory microenvironment, and produce an abscopal effect (102). These mechanisms are the fundamental basis for Nano-based RT-enhanced immunotherapy. The abscopal effect, a systemic inhibitory effect on metastatic tumors beyond the radiation field, was first discovered in 1953 as a result of radiation therapy (103). To maximize the benefits of RT in systemic therapy, scientists are actively exploring drugs and delivery methods that are compatible with RT. For example, cisplatin is said to increase the CD8+ T cells in RT plus anti-PD-1 treated tumors. Ying Wang et al. then explored that cisplatin (CDDP) loaded complex nanoparticles consisting of poly (L-glutamic acid)-graft-methoxy poly (ethylene glycol) (CDDP-NPs) have a stronger inhibitory effect on RT and amplify RT-induced ICD in models of Lewis lung carcinoma. Furthermore, CDDP-NPs can significantly improve the abscopal effect of RT plus anti-PD-1 in the treatment of non-radiotherapy tumors (64). This promising combination strategy was reported by another study. The efficacy of radioimmunotherapy for colorectal cancer is limited. While “carrier free” coordination polymer nanorods (ZGd-NRs) (composed of zoledronic acid and gadolinium) have been shown to deposit X-rays, enhance ROS induced by RT, and induce ICD characterized by increased CRT, HMGB1, and ATP expression in CT26 cell lines. Additionally, ZGd-NRs improves the immune-suppressive microenvironment by inhibiting TAMs and promoting DC maturation and ZGd-NRs sensitized radiotherapy can significantly suppress distant tumor growth. This effect is further amplified by PD-L1 blockers (66). In clinical practice, RT-induced ICD has been limited to only inducing modest levels of ICD. Therefore, Wang et al. constructed a Cu-based mixed-valent (Cu+/Cu2+) nanoscale coordination polymer (Cu-NCPs) that can generate hydroxyl radicals and deplete GSH, thus enhancing the generation of RT-induced ICD (104). In recent years, Gold nanoparticles (AuNPs) have been used extensively in radiotherapy sensitization studies, which can catalyze the generation of free radicals and very low-energy electrons to enhance the sensitivity of DNA to ionizing radiation on the one hand, and enhance radiation damage effects through oxidative stress, cell cycle blockade, and inhibition of DNA repair on the other hand (105–107). It’s true that AuNPs can enhance the efficacy of cancer radiotherapy by absorbing radiation energy locally in tumors, while protecting surrounding normal tissues from radiation toxicity. Branislava Janic et al. found that 14-nm AuNPs significantly enhance RT-induced ICD and increase macrophage infiltration in tumor tissue. The delayed tumor growth and improved overall survival revealed additional underlying immunological mechanisms and provided a platform for studying a multimodal approach to RT in triple negative breast cancer (65).

PDT is a therapeutic modality based on photosensitizers (PS) and oxygen within the tumor tissue. After selective accumulation of PS in the tumor, appropriate wavelengths of visible light are used to excite the PS. This leads to the production of ROS within the tumor, resulting in cell death and destruction of the tumor tissue (108). This non-invasive treatment produces fewer side effects. To enhance the efficacy of PDT, emerging strategies include hypoxic reversal nanomedicine and multi-functional photosensitizers (109). For instance, Huang Cong et al. designed a nanoparticle CaO2@CuS-MnO2@HA (CCMH) for breast cancer therapy. CaO2 reacts with H2O to produce a large amount of oxygen, which promotes CuS-mediated PDT, leading to ICD. *In vivo* experiments combining anti-PD-L1 and CCMH effectively increased the matured DCs, M1 macrophages, and CD8+ T cells in tumor tissue, indicating it a promising treatment (110). For another, a rational design of photosensitizer-based nanoplatform (CAM-NPs) was constructed with the help of human serum albumin (HSA), composed of chlorin e6 (Ce6, a photosensitizer), axitinib (AXT, a tyrosine kinase inhibitor) and dextro-1-methyl tryptophan (1MT, an IDO inhibitor). CAM-NPs treated cells after laser irradiation both secreted significant ICD biomarkers (CRT, ATP and HMGB1) and reverse the hypoxic situation, which proved its excellent performance for immunotherapy (111). The regulation of TME greatly enhances immunotherapy. LIC, a multifunctional nanodrug encapsulating IPI-549 (a PI3Kγ inhibitor to inhibit MDSCs) and Ce6, can penetrate CT26 cells to induce ROS and ICD production. Additionally, LIC reprograms the TIME by decreasing the amount of MDSCs, Tregs, and M2-TAMs as well as increasing the maturity of DC and the infiltration of CD8+ T cells (112). Smart nanoparticles provide convenience to drug delivery. PH-dependent smart nanomaterials (M(a)D@PI-PEG-RGD) are proportionally loaded with doxorubicin, NH_4_HCO_3_, and PS while highly targeting the RGD modification. The decomposition of NH4HCO3 stimulated by PH into bubbles hastens the doxorubicin’s release. Additionally, the combination of M(a)D@PI-PEG-RGD and PDT significantly inhibits tumor growth, it provides exploration value for immunotherapy research (113). More importantly, photosensitizers with fluorescent emission enable molecular image-guided PDT/immunotherapy. This integrated approach to diagnosis and treatment has drawn a lot of attention for its time and cost-effectiveness. Liu Qiang et al. designed self-assembled nanomaterials that encapsulated photosensitized agent BDP-I-N and anti-PD-L1 drugs. Immunotherapy guidance can be done through real-time imaging of PD-L1 immune checkpoint in the NIR II window (1000-1700nm). These NPs produced singlet oxygen to induce tumor elimination, and no evident toxicity was observed in mice with MC38 tumors (114). Several powerful fluorescent photosensitizers, such as porphyrins (115), aggregation-induced emission (AIE) (116), and iodinated cyanine dyes (117), have demonstrated satisfactory performance in mouse models. Multimodal therapy is necessary when needed. For instance, a multifunctional nanoplatform that utilizes single aggregation-induced emission luminogen (AIEgen) and EPR effects is capable of performing image-guided surgery-PTT/PDT, while the use of PD-L1 antibodies produces a satisfactory immunotherapy effect. During the treatment, AIEgen generates ROS, which helps to facilitate the ICD process (118). More recently, an interesting research found that PDT-induced P53 can reprogram M2 TAM into M1 TAM, providing us with new ideas for PDT-enhanced tumor immunity (119).

PTT is a method of selectively ablating tumors through the conversion of near-infrared (NIR) laser energy into heat therapy using a photothermal agent. However, tumor recurrence and metastasis are often associated with unequal heat distribution on the tumor surface and tumor immunosuppressive microenvironment (78). NDDS use their advantages to break these limitations. Zhaowei Li et al. designed a multifunctional biomimetic nanoplatform called AuDRM with both pH and temperature stimulation response characteristics. Their results showed that laser irradiation can induce ICD through PTT by increasing the tumor temperature. In addition, the release of R837, an immunostimulant, and tumor antigen from the nanomaterial enhanced immunotherapeutic effects and long-term immunological memory. Furthermore, the shedding of pH-sensitive membranes increases exposure to gold nanoparticles, effectively releasing R837 to enhance immunotherapy (79). HA-AuNR/M-M2pep NP, which is a gold nanorod modified with hyaluronic acid (HA), can cleave the MMP2-sensitive peptide on the surface of tumor cells, leading to the release of M2pep, the selective consumption of M2-TAM, the recruitment of TILs, the activation of T cells, and the secretion of anti-tumor cytokines (like IFN-γ and TNF-α). Moreover, HA exhibits excellent biocompatibility, biodegradability, and CD44 receptor binding affinity. HA-AuNR accurately targets tumor tissues (because the tumor cells express CD44 at a high level) under NIR laser for PTT, stimulating the ICD of tumor cells and anti-tumor immunity (77). This EPR effect is effective in several cancer models. After HA modification, black phosphorus (BP) acts as a PTT/PDT reagent exhibiting strong electrical conductivity, strong optical properties, and low toxicity, boosting the EPR effect at tumor sites. Regarding *in vitro* and *in vivo* experiments based on 4T1 cell lines, the HA-BP granules exhibited exceptional imaging capability and PTT treatment efficiency. Additionally, HA-BP can induce M2-to-M1 polarization of macrophages, leading to the induction of ICD (70). Nevertheless, obtaining complete tumor remission through PTT/PDT alone is challenging. Hence, the combination of nano-based PTT/PDT with other treatments, mainly immunotherapy, is regarded as having better clinical prospects. For example, Liangjie Jin’s team has designed a corn-like Au/Ag nanorod (NR) that can provoke tumor ICD at the NIR-II window (1064 nm), increase tumor T cells infiltration significantly, and change ‘cold’ tumors into ‘hot’ ones. The combination of CTLA4 antibody and Au/Ag-NRs-based PTT/PDT can generate a robust immune memory effect, preventing the recurrence of breast tumors and distant metastasis (120). The similar combination also proved perfect clinical effect in TNBC (80).

SDT is an emerging non-invasive and deep tissue penetrating therapeutic method with great potential in the treatment of tumors. SDT mainly kills tumor cells by producing ROS through ultrasound waves (121). Currently, small molecule sonosensitizers face issues such as poor stability and ROS generation performance. To improve the therapeutic effects of sonosensitizers, researchers have developed several nano-sonosensitizers, including Au-MnO and Au-TiO2 (122–124). Nevertheless, these sonosensitizers have low efficiency in killing tumor cells, exhibit poor tissue selectivity due to their sustained pharmacological activity, and may cause off-target toxicity. Future explorations should focus on multifunctional nanomaterials and novel sonosensitizers. IRO@FA NPs, a complex sonosensitizer nanoparticle, has a perfluorohexane core and shells of IR780, PLGA, PEG, and FA (PLGA: poly (lactic-co-glycolic) acid; PEG: polyethylene glycol; FA: folate). IRO@FA NPs generate enough ROS upon ultrasound irradiation and produce DAMPs, inducing ICD in ID8 ovarian cancer cells. *In vivo* results revealed that IRO@FA NP-mediated SDT caused the infiltration of CD3+ T and CD8+ T cells, leading to a significant up-regulation of PD-L1 expression, potentially assisting ICI treatment (81). Xuan Tan’s research team designed a novel core-shell transformable nano sonosensitizer, TiO2@CaP. TiO2@CaP significantly enhanced ICD, T-cell recruitment and infiltration, and transforms immunogenic ‘cold’ tumors into ‘hot’ tumors. In combination with anti-PD-1, TiO2@CaP-mediated sonodynamic therapy suppressed lung metastases and untreated distant tumor growth (125).

In conclusion, nanomaterials play a powerful role as novel drug delivery systems in ablative therapy (RT, PDT, PTT and SDT etc.). Taking full advantage of the physical properties and easy design properties of nanomaterials can enhance ICD production and significantly increase the immune response. Finally, the combination of ICIs can further enhance the efficacy of systemic therapy and reduce the toxic side effects, tumor recurrence and metastasis.

#### 4.1.3 Nano-based gene editing

Gene editing technology permits precise regulation of target gene expression in immune cells, boosting the immune response. The nano drug delivery system is the current most promising carrier, circumventing the instability and large size of gene editing products and accurately delivering target genes to cells (126). Clustered regularly interspaced short palindromic repeat-associated nuclease 9 (CRISPR/cas9) and small interfering RNA (siRNA) frequently intercept the expression of immune-regulating genes. Initially, IDO1 siRNA-based NPs reversed IDO1-induced immunosuppression and strengthened the ICD-induced immune response. This tumor-fighting approach demonstrated efficacy in colorectal and orthotopic pancreatic tumor models (84). Menghao Shi et al. designed a novel charge-switchable, acid-triggered nanoparticle incorporating IDO1 siRNA and mitoxantrone in 2022. Under the acidity conditions of TME, the drug is released efficiently, which promotes the maturation of DC cells, improves the infiltration of CTLs and down-regulates the number of Tregs, and finally causes strong anti-tumor immune response (85). Conversely, SDT sound-sensitive agents have the potential to generate ROS and contribute to ICD. However, the naturally occurring REDOX regulatory pathway in tumor cells can weaken the effect of ROS and other harmful substances and thus develop resistance to SDT therapy. The classical deoxidation signaling pathway mediated by nuclear factor (erythroid-derived 2)-like 2 (Nrf2) was discovered to cause tumor cell resistance to PDT or SDT through persistent ROS consumption (127). Therefore, we hypothesize that Nrf2 knockout can reverse this resistance phenomenon. In 2021, TIR@siRNA was designed to deliver Nrf2-siRNA into the cytoplasm of CT26 cells, inducing the downregulation of Nrf2, DNA damage, and cell apoptosis, significantly improving SDT cytotoxicity and ICD induction. *In vivo* experiments, the use of TIR@siRNA greatly enhanced the antitumor effect of SDT and even stimulated the tumor immune response to enhance PD-L1 therapy in patients (86). In several laboratories, nanomaterials have been employed in the most popular chemo-immunotherapy regimen for many solid tumors to deliver PD-L1 knockout genes and chemotherapy drugs, demonstrating satisfactory efficacy. Ling Xiang et al. examined a type of nanoscale coordination polymer particles that, through the property of low-pH spurt inducing excessive osmotic pressure in endo/lysosomes, effectively released PD-L1 siRNA, carboplatin, and digitoxin into the cytoplasm. The expression of ICD markers (CRT, Hsp70) and the percentages of CD3ϵ+CD4+ Helper T cells (Ths) and CD3ϵ+CD8a+ Cytotoxic T cells (Tcs) were notably increased with triple therapy, suggesting a possible combination therapy for advanced and aggressive tumors (87).

### 4.2 NDDS enhances immunotherapy by regulating TIME in an ICD-independent way

**Figure 5:**
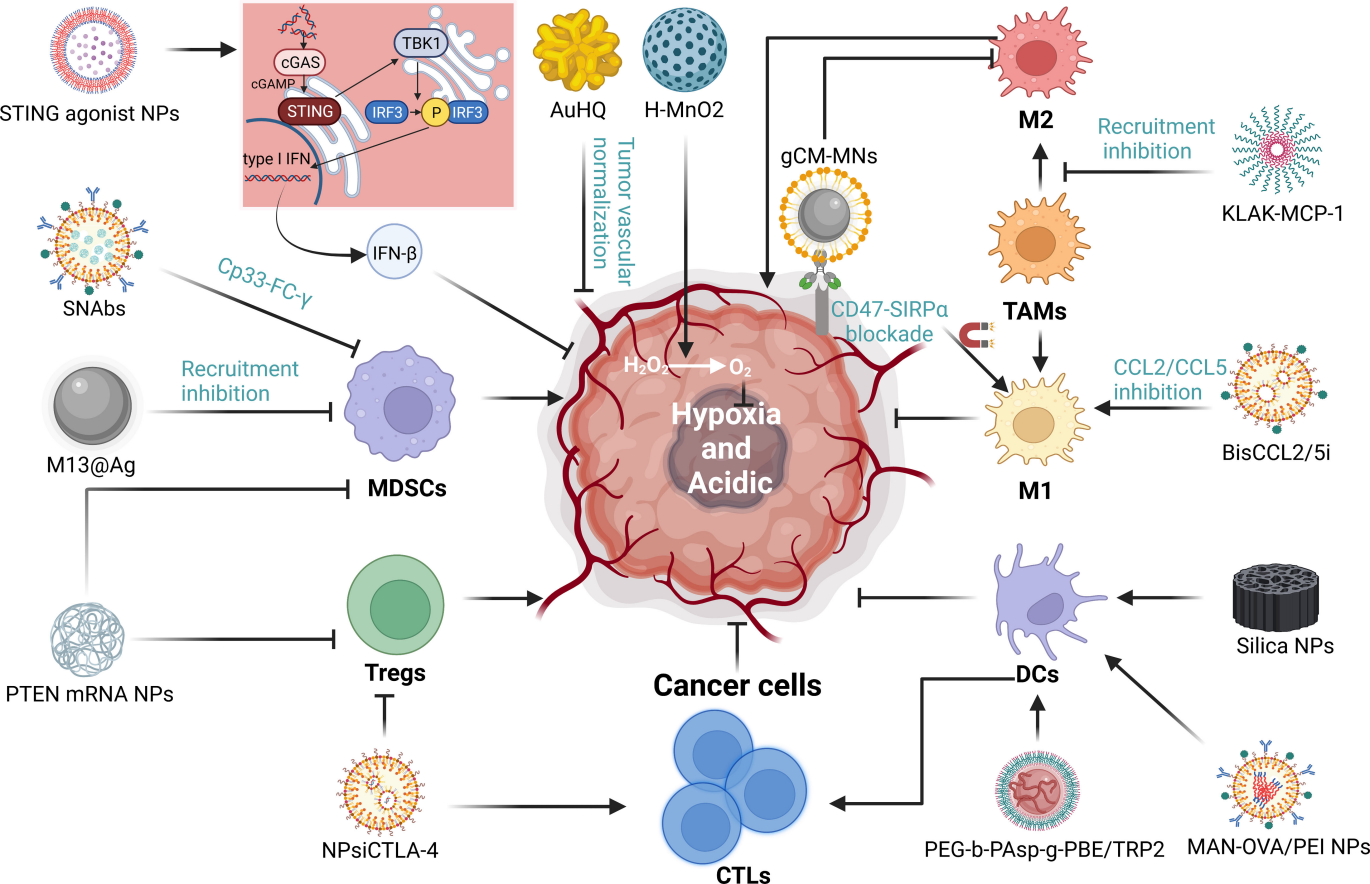
The mechanisms of NDDS regulating tumor immune microenvironment (TIME). We summarized the mechanisms by which nanoparticles improve the immunosuppressive microenvironment through targeting immune cells, hypoxia and and acidity environment and significant signaling. “→” represents the pointing and promoting effect. “⊥” represents the inhibiting effect.

The TIME is a complex milieu of competing immune promotion and suppression. In many tumors, the recruitment of immunosuppressive cells such as MDSCs, TAMs, and Tregs while diminishing the proportion of essential immune cells such as CTLs, NKs, and DCs creates a TIME that ultimately leads to resistance to immunotherapy (128). Furthermore, hypoxia and acidic environments are also major obstacles to immunotherapy. We have already discussed several NDDS-based strategies to improve immunotherapy primarily by inducing ICD, but also by improving the immune microenvironment. In fact, reversing the immunosuppressive microenvironment is indeed very important for immunotherapy. Next, we will discuss NDDS-based strategies for reversing immunosuppression independent of ICD process. It mainly includes: 1) enhance the infiltration of immune cells; 2) reduce the number and function of immunosuppressive cells; 3) improve hypoxic environment; 4) activate important immune-related signaling pathways (**Figure 5**).

#### 4.2.1 Immune cells

MDSCs are heterogeneous and immature cells of bone marrow origin that can be found in almost all types of tumors and malignant or pathological conditions (e.g., infections, autoimmune diseases, and trauma). Within tumors, MDSCs are triggered by pro-inflammatory mediators that inhibit T-cell activation and encourage tumor immune tolerance and growth (129). Nanodelivery systems can induce significant changes in the number and function of MDSCs, reducing their immunosuppressive effects. Some studies suggest that the loss of phosphatase and tensin homolog (PTEN) leads to the accumulation of MDSCs and Tregs (130). In one study, a polymeric nanoparticle encapsulating PTEN mRNA showed promise in both melanoma and prostate cancer models, where it was able to restore PTEN function in cancer cells, leading to the observed reduction in MDSCs and Tregs numbers (131). Recent studies have indicated that microbiota influences tumor immunology. Specifically, Fusobacterium nucleatum (Fn), commonly found in colorectal cancer, selectively amplifies MDSCs (132). To inhibit Fn function, silver nanoparticles (AgNP) were electrostatically assembled around the Fn-binding M13 phage surface capsid protein (M13@Ag) to achieve a specific clearance of Fn, ultimately reducing MDSC numbers. Additionally, M13@Ag nanoparticles also stimulated antigen-presenting cells (APCs) to further activate the host immune response (133). Impressively, circulating MDSCs in a TNBC mouse model were completely depleted by Synthetic Nanoparticle Antibodies (SNAbs). SNAbs are engineered with cp33 peptide, a human IgG1 Fc mimicking ligand that binds to FC-γ receptors (FC-γ rs) on immune effector cells and can precisely target MDSCs (134).

TAMs, one of the most abundant immune cell populations in the TME, display heterogeneity and differentiation plasticity, and can transit between anti-tumor and pro-tumor states. M1 TAMs are known to be anti-tumoral while M2 TAMs are pro-tumoral (135). TAMs play a significant role in various malignant solid tumors such as breast, prostate, liver, lung, ovarian, gastric, pancreatic, and colorectal cancers. Notably, the presence of M2 is associated with a poorer prognosis for tumors (136–140). Numerous studies found that nanoparticles can efficiently limit TAMs survival and recruitment, and repolarize M2 into the M1 (141). For instance, a lipid NPs encapsulating mRNA encoding CCL2/CCL5 inhibitor reversed immune suppression in liver cancer by inducing M1 polarization. Moreover, in conjunction with anti-PD-L1, it significantly improved the survival in mice with primary hepatocellular, colorectal, and pancreatic cancers with liver metastasis (142). Gene editing nanoparticles also offer novel ideas (143). Cancer cells express CD47, which binds to a signal-regulatory protein α (SiRP-α) receptor on macrophages to protect them from phagocytosis (144, 145). Lang Rao’s team designed genetically edited cell membrane-coated magnetic nanoparticles (gCM-MNs). The gCM shell overexpresses the SIRPα variant at the gene level with a 50,000-fold enhanced affinity for CD47, effectively blocking the CD47-SIRPα signaling pathway, while the MN nucleus promotes M2 to M1 repolarization, restores macrophage phagocytosis of tumor cells and triggers antitumor T cells immunity (146). Recently, a KLAK-MCP-1 micelle composed of CCR2-targeted peptide sequence and apoptosis-inducing KLAK peptide inhibited melanoma growth in B16F10 mouse models by inhibiting MCP-1/CCR2 axis and decreasing the recruitment of TAMs (147). Although numerous studies have demonstrated that nanoparticles can enhance anti-tumor efficacy, their use has not yet been widely used in clinical settings, and further clinical trials are necessary to establish their safety and efficacy in humans.

Tregs can suppress aberrant immune responses against autoantigens and anti-tumor immunity (148). Tregs infiltrating tumors have been detected in different types of cancer, such as pancreatic, liver, gastrointestinal, and lung cancers. Their abundance is often related to a poor clinical prognosis (149, 150). Recent studies have provided increasing evidence that blocking or eliminating Tregs cells promotes anti-tumor immune responses (151, 152). Immunotherapy can successfully target Tregs through specific molecules like CTLA-4, GITR, CCR4, PD-1, OX-40, and LAG3 (148). In a subset of cancer patients with a poor prognosis, the inhibition of PD-1 and CTLA-4 has increased their progression-free survival (153). Nanoparticles are being developed using NDDS technology to precisely target Tregs. Early in 2016, Li et al. have designed a nanoparticle (NPsiCTLA-4) that could distribute the CTLA-4-siRNA to the CD4+ and CD8+ T cell subsets present in the tumor microenvironment. Their study demonstrated a significant increase in the percentage of CD8+ T cells, which led to a decrease in the number of Tregs in tumor-infiltrating lymphocytes (TIL) (154). Future investigations on immunotherapy approaches to target Tregs with nanoparticles hold significant potential.

DCs play a crucial role in regulating the adaptive immune response and are vital for T-cell-mediated immunity against cancer. Tumor-associated conventional dendritic cells (cDCs) are responsible for endocytosing dead tumor cells or cellular debris and transporting cancer-associated antigens to draining lymph nodes, where T-cell initiation and activation occur (155, 156). DC vaccines are often used to enhance the anti-tumor ability of the immune system by increasing the number of DCS in TME and promoting DCs antigen presentation. Nevertheless, various technical problems impede the delivery of vaccine antigens to DCs, which limit the effectiveness of therapeutic interventions due to antigen uptake and presentation insufficiency by antigen-presenting cells (157–159). NPs exhibit tremendous potential as delivery systems for cancer vaccines by facilitating the co-delivery of tumor-associated antigens and adjuvants to DCs. Mesoporous silica nanoparticles with extra-large pores have been shown to stimulate DC activation and enhance antigen presentation, increase the secretion of pro-inflammatory cytokines, and inhibit tumor growth in both *in vitro* and *in vivo* studies (158). Pei et al. designed Mannose-functionalized antigen nanoparticles (MAN-OVA/PEI NPs) with the ability to escape the endosome for targeting DCs. Their study shows that MAN-OVA/PEI NPs significantly improved antigen uptake by DCs, caused cytoplasmic antigen release, and stimulated cytokine production and DC maturation considerably *in vitro* (159). Wang et al. developed a PBE (Phenyl Borate)-modified TRP2 nanovaccine that infiltrates the lymph nodes, absorbed by DCs, and triggers DCs maturation. Unlike conventional cancer vaccines, the TRP2 nanovaccine also overcomes standard cancer vaccines’ challenges and stimulates the body’s most potent T-cell immune response to melanoma without an external adjuvant or antitumor activity (160).

#### 4.2.2 Targeting Hypoxia and acidity

The tumor microenvironment is characterized by hypoxia and acidity, which are attributed to the rapid proliferation of tumor cells, increased metabolic rate, and inadequate blood supply (161, 162). Hypoxia frequently leads to therapeutic resistance, including immunotherapy (163),chemotherapy (164), RT (165), PDT (166) etc. Multifunctional nanomaterials can enhance oxygen-dependent therapies such as RT, PDT and SDT by improving the anoxic and acidic microenvironment of tumors, which have been discussed in section 4.1. MnO2-based nanomaterials to improve hypoxia are the most frequently used in research (167, 168). However, in some tumors, increasing the O2 concentration alone is not enough, because the antioxidant substances (e.g., GSH) present in cancer cells also consume oxygen. Therefore, on the one hand, nanoparticles made of MnO2 can neutralize GSH with their own oxidative properties, and on the other hand, co-delivery of Bcl-2 inhibitors can contribute to reduce GSH (169). Lastly, tumor vascular normalization has emerged as a novel strategy for enhancing tumor immunity by reducing hypoxia and increasing perfusion (170). Wang and colleagues developed a protocol to reduce the expression of angiopoietin-2, VEGF, and bFGF in human umbilical vein endothelial cells using 8-hydroxyquinoline-modified gold nanomaterials (AuHQ), which inhibited the production of ROS in tumor cells by chelating iron ions. *In vivo*, AuHQ regulates tumor leakage, reduces tumor hypoxia, and increases blood perfusion, thereby inducing normalization of tumor vasculature (171). Despite the challenges faced by nanotechnologies regarding their safety and the stability of their effects, they possess great potential and merit continued attention and exploration.

#### 4.2.3 Significant signaling

Stimulator of interferon genes (STING) signaling is a promising target for tumor immunotherapy. STING was discovered as a crucial molecule in the innate immune response in 2008 (172). The cGAS-STING pathway resides in the endoplasmic reticulum. Upon receiving exogenous DNA, cGAS produces cyclic dinucleotides (CDNs) that activate STING. This leads to the formation of a tetramer that recruits TANK-binding kinase 1 (TBK1) proteins to Golgi, causing TBK1 to phosphorylate IRF3 and inducing the production of type I interferon (especially IFN-β). This process also activates multiple pro-inflammatory factors and chemokines (such as CXCL10 and CCL5), which attract T cells and natural killer cells (173). As a result, STING activators have been developed to regulate the immune microenvironment and achieve anti-cancer effects. At present, cyclic dinucleotides (ADU-S100, MK-1454) (174, 175) and macrocyclic bridge drugs (E7766) (NCT04144140) have entered clinical studies, but they are associated with significant transportation issues and side effects. This problem can be solved by NDDS technology (176). Compared with non-NPs administration, nanomaterial encapsulated drugs increased the half-life by 40 times, took advantage of EPR effect to accumulate drug concentration in the tumor, and increased the number of immune cells (CD4+ and CD8+ T cells) by 20 times. In addition, combined PD-1/PD-L1 drugs significantly reduced tumor burden in melanoma and breast cancer (177). Not only that, multifunctional nanomaterials and new therapeutic strategies are also under investigation. For example, Su Ting et al. designed a PH-responsive nanovaccine that delivers both tumor-specific antigen and STING agonist, selectively generating IFN response, enhancing DCs antigen presentation and sustained T cells response (178). Notably, Mn2+ is said to increase the STING agonist effect by 12-77 times. Their self-assembled nanoparticles effectively deliver them to tumor tissues, exerting powerful anti-tumor immunity, suggesting the potential of combining nanomedical and metal immunotherapy (179). More recently, some researchers have abandoned STING agonists because of its poor efficacy. Instead, a method was proposed to activate STING pathway *in situ* using nanoparticles to deliver DNA-targeted chemotherapy drugs (SN38-NPs). SN38-NPs causes DNA damage and leakage within tumor cells, which is transmitted from tumor cells to DCs via DNA-containing exosomes and subsequently activates the STING pathway. This therapy significantly reduced the toxicity of free SN38 and increased the rate of tumor suppression (80%) (180). Nanomaterial delivery systems have broad clinical application prospects for the activation of signaling pathways.

## 6 Conclusion and prospects

For the treatment of solid tumors, immune checkpoint inhibitors, targeted drugs, chemotherapy drugs, ablative surgery and their combination therapy are the current mainstream. Among them, immunotherapy is the paramount. However, treatment resistance and severe side effects often occur. To address these issues, we have found that changing drug delivery routes is effective. For example, nano-drug delivery systems can enhance immune efficacy through a variety of advantages, including enhancing drug permeability and retention in tumors, improving the solubility of hydrophobic drugs, targeting drug delivery, reducing physiological barriers to drug delivery, achieving integration of diagnosis and treatment, and providing multi-functional intelligent nanoplatforms. A number of preclinical studies have found that nanoparticles enhance immune efficacy mainly by inducing tumor immunogenic cell death and improving the immunosuppressive microenvironment. Moreover, most nanoparticles-based therapies combined with immune checkpoint inhibitors tend to have better anti-tumor effects in mice. However, unfortunately, most of these successful cases have only been realized in animal trials and rarely applied to the clinic. In the future, the fine application of nanomedicine delivery systems in the clinic still faces great challenges.

## Author contributions

YQ and HH designed and reviewed the article; JZ and SW were responsible for writing the article and drawing pictures; JZ was also responsible for the revision of the article. DZ summarized the tables, and XH and XW were responsible for reviewing the article. All authors contributed to the article and approved the submitted version.
